# Actions for addiction—Century-old concepts as possible game changers in addiction research, intervention, societal views, and policymaking

**DOI:** 10.1371/journal.pmen.0000030

**Published:** 2024-06-04

**Authors:** Christian Beste

**Affiliations:** 1 Cognitive Neurophysiology, Department of Child and Adolescent Psychiatry, Faculty of Medicine, TU Dresden, Dresden, Germany; 2 University Neuropsychology Center, Faculty of Medicine, TU Dresden, Dresden, Germany; PLOS: Public Library of Science, UNITED KINGDOM

Millions of people suffer from substance use disorders (SUDs) and billions of dollars are spent for research and treatment on this [1]. SUDs refer to the use of substances leading to significant impairment or distress. Despite many research efforts, a consensual concept covering the nature of SUDs is lacking and the effectiveness of cognitive-behavioral interventions is often small [2], with evidence for their value as an add-ons to pharmacotherapy being mixed [3].

Here, I argue that this is due to a myopia of what addiction reflects in its core–a *self-initiated action that is persistently pursued*. Regardless of the specific SUD, everything that leads to addiction starts with a single action (e.g., lighting a cigarette). Importantly, at the time of this first action, current explanations of addictions are not applicable (e.g., reinforcement learning concepts, reward concepts, biopsychosocial models etc.) as they rest on mechanisms that explain (addictive) behaviours once they are established and the first rewarding experience has been made. So, the elephant in the room is not why addictive behaviour is maintained, but why a self-initiated action is commenced that can ultimately lead to an addiction? It is well-known that substances themselves directly contribute to physical dependence. However, what also needs to be prioritized in research is why addictions sometimes emerge. In other words: *(i) Why are people trying things that can lead to addiction*? *(ii) Does an answer to that question provide alternative views on the strategy of interventions*? Attempts to answer these questions require a shift in research priorities and substance policies as well as less stigmatising societal views and media portrayals of addiction. This necessitates in shift in the conceptual starting point to frame SUDs.

Current foci of addiction research deal with aspects when the first major step towards an addiction has already been taken. This is at least partly explained by the fact that treatments are mostly commenced once detrimental effects of addiction are evident. A cognitive science framing of human action could help to avoid such problems is ideomotor theory. Ideomotor theory [4] explains how agents establish links between perception and action. While this theory is discussed in other fields of research, it is mostly neglected in the field of addiction. However, I propose that it has much to offer for addiction research as it assumes that an anticipation of a perceptual action effect is fundamental to initiating an action. Only thereafter, actions can become associated with specific thoughts or stimuli, leading to more readily occurring, potentially rewarding actions. Ideomotor theory suggests that bodily movements (including goal-directed actions) can be influenced by thoughts, ideas, or mental imagery without conscious awareness. When a person entertains a thought, it can trigger automatic, involuntary actions related to that thought. In other words, ideomotor theory assumes that actions are represented by their effects and any effect image, either endogenously or exogenously, will trigger the corresponding action. Unlike concepts dominating addiction research, ideomotor concepts can frame *why* a first action, ultimately leading to addiction, is commenced. Yet, another aspect of ideomotor concepts is even more radical concerning addictions: ideomotor concepts discard binary dimensions of controlled behaviour implying that there is some form of behavioural automaticity [5]. Many lines of evidence driven by ideomotor theory show that this dichotomy of thinking is ill-defined [5, 6] and that approaches to use the criterion of reward sensitivity and rationality to differentiate between controlled and automatic action are not viable [6]. Nevertheless, the dichotomy of controlled and automated processes is influencial in addiction research [7, 8] and the conceptual backbone of behavioural interventions [9]. Generally, a common theme in the clinician’s view of addictions is that controlled processes must be strengthened in people with SUD to regain control over drug intake–especially when drug use is chronic. However, this is questionable from a cognitive science (ideomotor) perspective and given psychiatry’s definition of SUDs. According to the American Psychiatric Association, “People with SUD have an intense focus on using a certain substance(s), to the point where the person’s ability to function in day-to-day life becomes impaired. People keep using the substance even when they know it is causing or will cause problems”. Thus, there is no generalized lack of control or willpower in SUDs. It is a matter of the severity. The critical point is that before an addiction becomes “uncontrollable” in late stages, there is a choice to engage with a behavior in the anticipation of an effect (cf. ideomotor principle). Initially, when SUDs are less severe, pursuing actions may reflect over-functioning willpower/control. Therefore, trying to increase control in SUDs is like fighting the devil with the Beelzebub- one is further increasing brain functions that are causing addictive behaviour maintenance. This, however, is still the nowadays goal of treatment efforts towards abstinence. Current interventions aim to train individuals to develop alternative behaviors that overcome the urges to use substances. This training of an alternative action/behavior is shaped by the mindset that there is some (automated) behavior that needs to be replaced through engaging control. An ideomotor shift in perspective offers a radically different view on addiction and on approaches for treatment:

Specifically, one should *not* try to induce abstinence. This would require dissociation from an anticipated action effect, which evidence suggests is difficult to achieve and of limited success [2, 10]. Rather, one should actively use the *abilities* of people with addiction and train them to achieve the goal of addictive behaviours in healthier and legal ways. Central for ideomotor concepts is that actions are executed if there is an expected sensory effect [4]. The latter is broadly defined and includes anticipated changes in the neural activity profile [11]. Central neurophysiological mechanisms underlying ideomotor principles [12] are also altered in addiction [13]. This knowledge offers the opportunity to re-channel abilities in a healthier way, training people to induce brain states that mimic the effects of a substance. In substance abuse, it is often the side-effect of the drug causing detrimental physical health effects. These side-effects do not usually prevent further usage. It is the anticipation of desirable effects, which influence substance intake and is thus a central ideomotor principle. A critical step to therefore take in research is how to read the brain’s response to a substance’ effects and use this to train users to induce this brain state without taking the substance. Evidence has shown that ideomotor principles are likely governed by a specific set of neurophysiological oscillatory dynamics and their interplay [12]. These ideomotor principles should also be evident regarding the anticipation of drug effects. Thus, one approach could be to make use of these principles in AI-powered brain-computer interfacing and decoding devices/technologies [14] to train people to induce a “high” without the substance. Through this, the ability and persistence of affected individuals is re-channeled in a different way and the negative (somatic) effects of addiction-related mechanisms are avoided. Perverting addiction-related mechanisms through brain decoding in this way takes advantage of the abilities of people with addictions, which will have at least two major consequences:

- It changes the motivation of people to commence and adhere to addiction treatments because the position of the individual is more positive and treatment principles are not working against the ability of individuals to persistently follow goal-directed behavior.
- It considerably changes societal views on addiction and bears the potential to change policy.

From the ideomotor perspective, addictive behaviour is not different from other behaviours that conform to societal conventions. It is not the goal that people intend to achieve that is problematic, but the path to it (i.e., substance use). There are other forms of addictive behaviours which are more positively viewed; for example, workaholism or extensive exercise. Both reflect instances of addictions, which, leverage a societal benefit by driving economic growth. What makes these “accepted addictions” different from “stigmatized addictions” like SUDs? From a purely ideomotor perspective they are different in the means but not in their goal pursued with the addictive manifestation. Should drug policy therefore intend to prohibit people from inducing action-effects? Probably not. Should drug policy prevent people from having devastating effects coming with action effects? Definitely yes. Yet, the differentiation of “means” and “effects” is not evident in addiction research and is a consequence of widely neglecting basic cognitive science concepts on how actions are formed. Through the advent of AI-based neurotechnology enabling ‘brain decoding of thoughts’ there is a chance to reshape how addiction research and treatments could be conducted. If one considers people with addiction as having the ability to readily build and sustain anticipated action effects, it makes more sense to recognize their ideomotor potential *before* it leads to problematic behaviour. Earlier interventions would need to identify people at risk of developing stable action-effect contingencies as defined by ideomotor theory. Through the use of neurophysiological principles in AI-powered brain-computer interfacing technologies, people could then be trained to induce their desired effects that are otherwise achieved by drug-intake.

Such an ideomotor shift in concepts allows a more nuanced approach to the socially relevant question of what addictions are, how they emerge, how they should be treated and viewed by society. At present, scientific efforts and policymaking are driven by a view in which addiction is foremost viewed as a moral failing or a lack of willpower. This is stigmatizing and fueled by biased (social) media contributions. This problematic view in society could be reduced by a radical ideomotor shift about when to intervene with people prone to developing addictive behaviors and use their ideomotor abilities to re-channel the way they achieve their desired states. Current drug policies manifest stigmatization: Therefore, concepts must guide us from a deficit-oriented to an ability-oriented view. The ideomotor shift in perspective outlined above would help to achieve this through changes in treatment, policy and societal views.
